# Efficient Layer-Wise *N*:*M* Sparse CNN Accelerator with Flexible SPEC: Sparse Processing Element Clusters

**DOI:** 10.3390/mi14030528

**Published:** 2023-02-24

**Authors:** Xiaoru Xie, Mingyu Zhu, Siyuan Lu, Zhongfeng Wang

**Affiliations:** School of Electronic Science and Engineering, Nanjing University, Nanjing 210023, China

**Keywords:** sparse, convolutional neural networks, hardware acceleration on neural networks, FPGA design

## Abstract

Recently, the layer-wise *N*:*M* fine-grained sparse neural network algorithm (i.e., every *M*-weights contains *N* non-zero values) has attracted tremendous attention, as it can effectively reduce the computational complexity with negligible accuracy loss. However, the speed-up potential of this algorithm will not be fully exploited if the right hardware support is lacking. In this work, we design an efficient accelerator for the *N*:*M* sparse convolutional neural networks (CNNs) with layer-wise sparse patterns. First, we analyze the performances of different processing element (PE) structures and extensions to construct the flexible PE architecture. Second, the variable sparse convolutional dimensions and sparse ratios are involved in the hardware design. With a sparse PE cluster (SPEC) design, the hardware can efficiently accelerate CNNs with the layer-wise *N*:*M* pattern. Finally, we employ the proposed SPEC into the CNN accelerator with flexible network-on-chip and specially designed dataflow. We implement hardware accelerators on Xilinx ZCU102 FPGA and Xilinx VCU118 FPGA and evaluate them with classical CNNs such as Alexnet, VGG-16, and ResNet-50. Compared with existing accelerators designed for structured and unstructured pruned networks, our design achieves the best performance in terms of power efficiency.

## 1. Introduction

Convolutional Neural Networks (CNNs) have shown excellent accuracy in computer vision tasks [1,2,3]. However, CNNs are much more complex in calculations compared with traditional algorithms. CNNs cannot be fully exploited for high processing latency or extreme power consumption when executed on CPUs or GPUs. Recently, domain-specific accelerator (DSA) designs for CNNs have attracted tremendous attention. They can achieve comparable latency compared to GPUs, equivalent power compared to CPUs [4], or relatively high speed on power-limited edge devices.

Apart from the design of dedicated hardware, model compression techniques such as pruning [5,6,7,8,9] and quantization can also help reduce computational latency and power consumption. After the pruning algorithm, CNN weights can be compressed to 10% of the original network with negligible accuracy loss [5], so the computational complexity can be significantly reduced. Network pruning can be divided into unstructured-pruning and structured pruning. The unstructured-pruning technique generates sparse masks based on the magnitude of the weight value, with no location information contained in the sparse pattern. Structured pruning zeroes regular blocks of weights by adding specific patterns during the pruning. Compared to the unstructured pruned networks, the structured pruned ones may have lower accuracy but better speedup due to the restricted sparse pattern [8].

Various sparse patterns are proposed to better balance the hardware speedup and accuracy. Cao et al. [10] and Zhou et al. [8] pruned a network with *N*:*M* sparse pattern, where the *N*:*M* sparsity indicates each bank has *M* continuous weights and *N* elements are kept after pruning. Compared to structured pruning patterns, such as filter-wise, channel-wise, and shape-wise, the *N*:*M* sparse pattern saves the coarse-grained structure and exploits the fine-grained sparsity to maintain accuracy in different tasks. However, the uniform pruning pattern across the network treats layers with non-uniform redundancy equally, leading to a sub-optimal solution. A layer-wise *N*:*M* pruning pattern is developed in [9] to find independent sparse patterns for each layer, where *N* can be selected from 1 to *M*. Sensitive network layers are pruned less to retain network accuracy, while more weights are zeroed in robust layers to achieve a higher compression ratio. As illustrated in [9], the layer-wise *N*:*M* pruned networks can achieve comparable accuracy with unstructured pruned ones.

There are works accelerating *N*:*M* pruned networks [11,12,13]. Nevertheless, the parallelism settings in [11,12] are connected with the non-zero configuration *N*. Under-utilization will occur in these accelerators when dealing with variable *N*-configurations. Thus, we develop an efficient processing element (PE) for accelerating the layer-wise *N*:*M* sparse pattern. The dedicated designed PE can be easily exploited in dense network acceleration architecture, ignoring the tiling and randomness brought by layer-wise *N*:*M* sparse pattern. To enlarge the design space of the pruning and acceleration, we also consider the pruning dimensions, i.e., kernel-wise, input-channel-wise, and output-channel-wise.

Based on the above ideas, this paper proposes acceleration architectures with flexible sparse PE clusters (SPEC) for CNN networks to process the layer-wise *N*:*M* sparse pattern with the help of hardware-algorithm co-optimization. The main contributions are briefly described as follows.

1. To better accelerate the layer-wise *N*:*M* sparse pattern, we analyze the effect of sparse dimensions and variable *N*-configurations on hardware deployment. Based on the analysis, a basic sparse-PE is proposed to enhance the hardware performance of *N*:*M* pruning networks.

2. Based on the basic sparse-PE, the SPEC with more flexibility and parallelism is proposed to accelerate the layer-wise *N*:*M* pruned networks efficiently. Dealing with different sparse dimensions, SPECs that support inner-product *N*:*M* sparse (I-SPEC), outer-product *N*:*M* sparse (O-SPEC), and the combinations (IO-SPEC) are elaborately developed.

3. The proposed SPECs are integrated into a dense hardware architecture, which integrates the flexible network-on-chip and the channel-first dataflow. The hardware architecture with I-SPEC (ISA), the hardware architecture with O-SPEC (OSA), and the hardware architecture with IO-SPEC (IOSA) are established and evaluated in this paper.

We perform the algorithm and hardware experiments to show the effectiveness of our methods. Alexnet, VGG-16, and ResNet-50 with different sparse dimensions are utilized on the ImageNet dataset [14] to show the effectiveness of the enlarged pruning space. For the hardware aspect, the ISA is implemented on Xilinx ZCU102 FPGA. The OSA and IOSA are evaluated on Xilinx VCU118 FPGA. The proposed I-SPEC and acceleration architecture ISA are proven to be effective with the best power efficiency.

The layout of this paper is demonstrated as follows. Section 2 lists the recent works focusing on pruning CNNs and shows the background of the pattern pruning algorithms and hardware acceleration. The co-analysis of hardware and algorithm on the layer-wise *N*:*M* sparse pattern is presented in Section 3. Section 4 elaborates on the details of various proposed SPECs and introduces the overall hardware architecture briefly. The experiments and comparisons are illustrated in Section 5.

## 2. Background

Neural network pruning can be roughly divided into two categories: unstructured pruning and structured pruning. In unstructured pruning algorithms, weights are pruned based on their magnitudes. For structured pruning algorithms, sparse masks with restricted locations are utilized, such as channel-wise, filter-wise, and shape-wise [15].

Hybrid patterns with coarse-grained structure and fine-grained randomness have been developed to balance the performance of hardware speedup and algorithm accuracy. Sparse patterns are limited inside each kernel in [7]. Lu et al. [16] partitioned weights into different groups and applied unified sparsity across groups. Works [6,17] have also proposed hybrid sparse patterns and developed acceleration architectures. Overall architectures accelerating hybrid sparse patterns are similar to the dense ones, with slight differences in data access. To process the fine-grained randomness, PEs are developed elaborately, aiming at accelerating operations with specific sparse patterns.

Recent works [10,12,13] are developed to accelerate the *N*:*M* sparse patterns. Cao et al. [10] and Fang et al. [12] proposed algorithm-hardware co-optimized frameworks for *N*:*M* sparse general matrix multiplication (GEMM) acceleration, which are utilized in long short-term memory (LSTM) and Transformer networks, respectively. The structured sparse tensor accelerator (S2TA) is proposed in [13] to exploit the dual-side sparsity of CNNs with *N*:*M* sparse pattern for weights and activations. In [12,13], variable *N*:*M* configurations have been considered in both algorithm designs, but the fixed *N*-configuration is exploited in both hardware designs. Thus, under-utilization will be incurred while applying the layer-wise *N*:*M* sparse pattern.

Thus, to better deploy the layer-wise *N*:*M* pruning networks, a dedicated hardware design is needed. The variable sparse ratio involves the compression encoding and storage of non-zero weights. Apart from the sparse ratio, the pruning dimension also connects with the hardware implementation. Our sparse CNN accelerator considers the variety of the sparse ratio and sparse dimension induced by the layer-wise *N*:*M* sparse pattern. Moreover, the *M*-tiling of a specific dimension may influence the allocation of convolutions. The parallelism and deployment of convolutions are also elaborated in our proposed PE.

## 3. Algorithm-Hardware Co-Analysis

The non-uniform *N*:*M* can be variable in an *N*-configuration or *M*-configuration, which will affect the development of both the algorithm and hardware. In [9], the variable *N* is applied in the proposed layer-wise *N*:*M* sparse pattern, which shows comparable accuracy with the unstructured sparse networks. Non-zero weights have more random distribution in *N*:*M* sparse pattern with variable *M*-configuration. The increasing randomness will enlarge the algorithm search space and increase the compression complexity of non-zero weights. Thus, variable *N* will be exploited in our layer-wise *N*:*M* sparse pattern.

Table 1 shows the accuracy of *N*:*M* pruned networks under different configurations. It can be seen that with equivalent sparsity, the network with a larger *M* in *N*:*M* sparse pattern can get better accuracy. The larger the *M* is, the lesser restriction is added to the pruning of weights. Thus, to balance the algorithm accuracy and hardware complexity, our PE is developed based on the layer-wise *N*:*M* sparse pattern with fixed *M*-configuration M=16.

**Figure 1:**
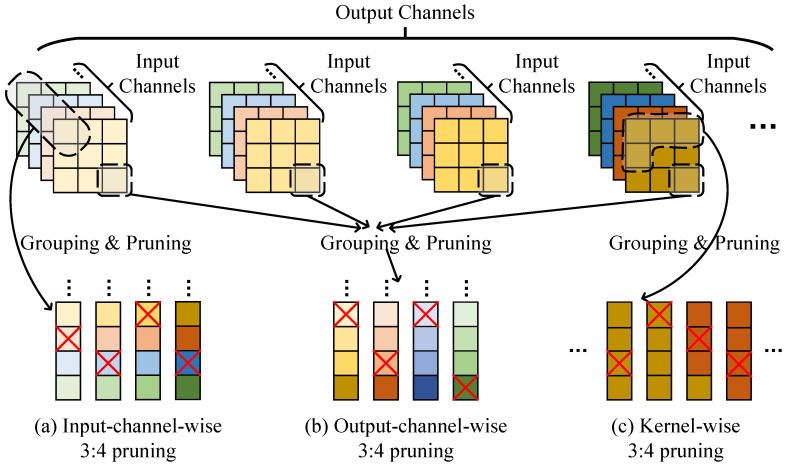
An example of convolution weights and 3:4 pruning in different convolutional dimensions.

As mentioned in Section 2, the sparse dimension will also affect the deployment of pruned algorithms. The pruning dimension can be categorized kernel-wise, input-channel-wise, and output-channel-wise based on the structure of the weight tensor. In Figure 1, there are 3:4 sparse patterns displayed with different pruning dimensions for the weight tensor. To perform the *N*:*M* pruning, three steps should be taken, which include flattening, grouping, and pruning. In the flattening step, the weight tensor is transformed into a one-dimensional vector with different transformations. The grouping step involves grouping adjacent *M* elements together as a block, where *M* is a predefined number. The pruning step then applies the *N*:*M* sparse pattern to each weight block. Only *N* weights are retained in each block after pruning. For pruning methods with different pruning dimensions, the flattening step is different, while the grouping and pruning steps are the same.

For input-channel-wise pruning, the weights with the same positions in different input channels in the same filter are flattened and converted into a one-dimensional vector. For output-channel-wise pruning, the weights with the same kernel position and input channel position in different filters are grouped together. Lastly, for kernel-wise pruning, the weight tensor is flattened along the kernel dimension and pruned accordingly. For input-channel-wise and kernel-wise sparse patterns, weights are pruned structurally across the inner production. However, typical kernel sizes (3×3, 5×5, 7×7, etc.) provide inadequate partition space for *N*:*M*. Im2col [18] conversion can be exploited in the kernel-wise *N*:*M* acceleration but introduces additional memory access. Output-channel-wise sparsity partitions weights across filters and prunes them with the *N*-configuration. The sparsity can be mapped regularly to an outer production. Channel-wise prunings have ample partition space and do not affect the reuse property of the convolution. The pruning dimension will not affect the sparse network accuracy, which will be shown in ablation experiments in Section 5.

**Figure 2:**
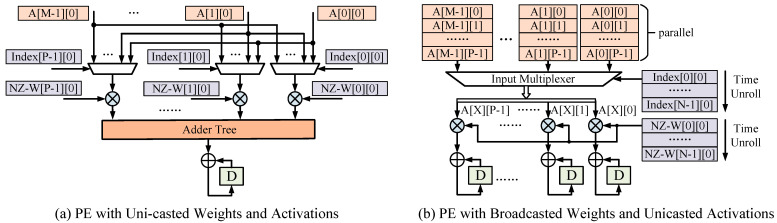
Processing Element (PE) architectures for input-channel-wise *N*:*M* sparse pattern. A[I][F] represents the F-th activation data in the I-th input feature plane. NZ-W[O][I] denotes the weights of the I-th input channel of the O-th output channel. The coordinates of Index[O][I] have the same meaning as NZ-W[O][I]. Symbols with ‘+’ and ‘x’ operators in circles denote adders and multipliers, respectively. These symbols have the same meaning in the rest of the article.

From the algorithm aspect, we focus on the hardware accelerator design with the non-uniform *N*:*16* channel-wise sparse pattern. Taking the I-SPEC for an example, Figure 2 illustrates some PE architectures processing *N*:*M* sparsity with the same multiplier accumulator (MAC) parallelism. For the PE in Figure 2a, element-wise multiplications with different non-zero weights are applied to MACs. This PE can be fully exploited only with fixed *N*:*M* sparsity and *N* being an integral multiple of *P*. In Figure 2b, input elements from the non-zero weight plane are deployed to MACs. The corresponding non-zero weight broadcasts to MACs and multiplies with input elements. The hardware efficiency of Figure 2b is not affected by the variable *N*-configuration for the parallel dimension, i.e., the feature map plane is orthogonal to the sparse dimension. The basic architecture for output-channel-wise pruning is dual to Figure 2b, where multiplexers controlled by indexes are responsible for selecting partial sum (PSUM). Based on the PE architecture with broadcasting non-zero weights, we propose hardware architectures that can support different *N*:*M* sparse patterns, which will be described in the subsequent sections.

## 4. Hardware Architecture for Layer-Wise *N*:*M* Sparse CNNs

Based on the basic PE architecture mentioned in Section 3, we propose the SPEC with more flexibility. In this section, the specifics of the SPEC and the integration will be illustrated in detail.

### 4.1. Flexible Sparse Processing-Element Clusters

**Figure 3:**
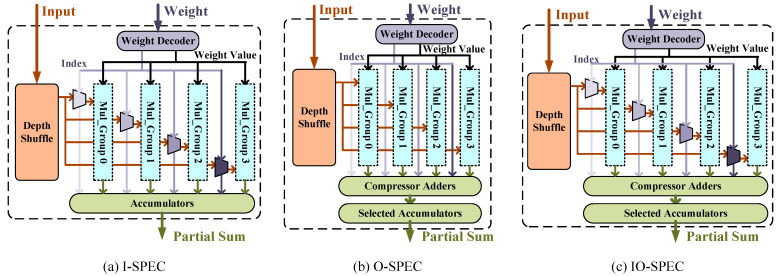
SPECs for different *N*:*M* sparse patterns. (**a**) presents the input-channel-wise sparse SPEC, i.e., I-SPEC. (**b**) denotes the output-channel-wise sparse SPEC, i.e., O-SPEC. (**c**) shows the SPEC architecture supporting both input-channel-wise and output-channel-wise sparsity.

With the accumulation of registers in Figure 2b, M×P inputs are only used once before being renewed. We also allocate *M* × *P* registers in PSUM accumulators to reuse inputs further. Thus, *M*-in *M*-out PSUMs are processed inside each PE, which we call the *MM*-tile. With sufficient local registers, each PE can be enlarged with more MACs; we call it a SPEC. The proposed SPEC is shown in Figure 3. Apart from the feature plane computation dimension illustrated in Section 3, intra-*M*-tile weight parallelism is also induced.

It can be seen from Figure 3 that MACs in each SPEC are partitioned as *G* identical groups, where *P* multipliers are allocated in each group. Each group processes data from the same feature plane and shares the same weight. *G* groups calculate parallel channels in the *MM*-tile, which are configured flexibly by controls generated from the Weight Decoder. Up to M×P inputs are renewed in the Depth Shuffle after each *MM*-tile processing. N×M cycles are occupied with processing each *MM*-tile. The minimum calculation time would be *M* cycles with N=1. Thus, we set the P=M to ensure the overlap between the transmission time for inputs and computation time, leading to the broadcast size of weight being *M* within each SPEC.

**Figure 4:**
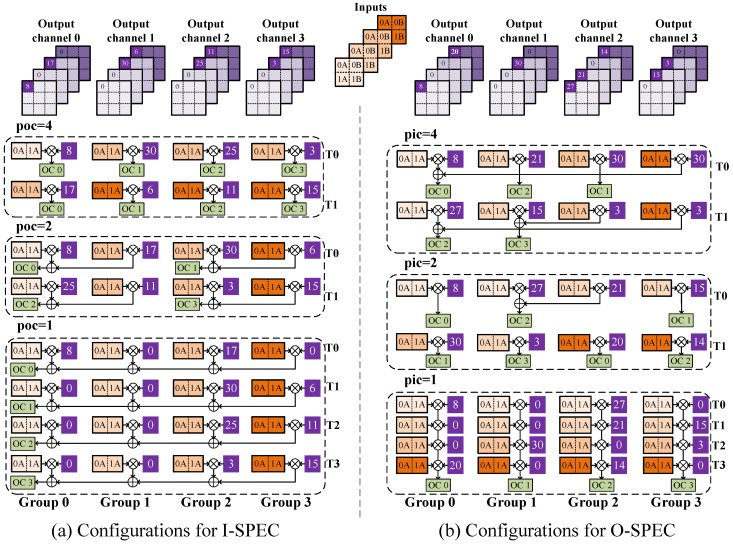
An example of SPECs with *G* = 4 under various configurations. The Tx in the figure denotes the processing cycles. Each multiplier illustrated as ‘x’ inside a circle denotes one group. (**a**,**b**) share the same inputs, which are shown in the top-middle of the figure and marked as Inputs. (**a**) shows three different configurations for I-SPEC. (**b**) shows three different configurations for O-SPEC.

Three types of SPECs processing different channel-wise sparse are illustrated in Figure 3. The I-SPEC, shown in Figure 3a, is utilized for the input-channel-wise *N*:*M* sparse pattern acceleration. Independent data selectors are inserted before input ports of multiple groups to support input channel sparse. The output ports of groups are connected to G×P PSUM accumulators in parallel. Various configurations of the I-SPEC are shown in Figure 4a, where poc is used to represent the output channel parallelism. When poc equals one, all groups are configured to process weights inside the *M*-tile of one filter. Inter-group accumulation is required, as shown in this figure. Groups would be idled with a sparsity where N is not the integral multiple of four. However, if poc equals four, no extra accumulations will be induced across groups, and groups will be fully exploited regardless of the sparsity configuration. Hence, with the poc control, each SPEC can extend the weight parallelism without decreasing hardware efficiency.

**Figure 5:**
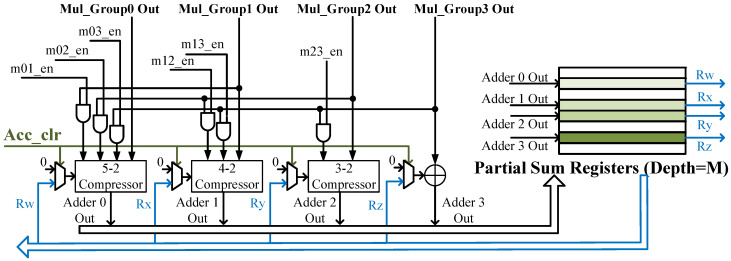
Details of the Compressor Adders and Selected Accumulators in the SPEC for output-channel-wise *N*:*M* sparse pattern.

Figure 3b is developed for the output channel *N*:*M* sparse pattern named the O-SPEC. It decodes indexes to control the PSUM processing. The processing details are shown as Figure 4b, where the pic denotes the input channel parallelism of the SPEC. With the fine-grained sparsity intra-*M*-tile, indexes may be identical across different groups. When processing with an input channel sparse, the independent multiplexer does not affect each other. Nevertheless, for output sparse, identical indexes lead to accumulation across groups. The problem is more complicated with the randomness intra-*M*-tile. To solve this problem, we add a module called Compressor Adders between MUL groups and accumulators to achieve the pre-addition before accumulation and induce input priority in the design accumulators. Figure 5 shows adders in detail. The m02_en is the signal that indicates whether the index of group0 and group2 are identical. If they are the same, the signal is pulled high to add the PSUM of group0 and group2. When writing back to the partial sum register, only Adder0 Out is enabled. The design of Figure 3c combines the aforementioned features and can support both input and output channel *N*:*M* sparse patterns, known as the IO-SPEC.

### 4.2. Weight Encoding and Decoding for the Layer-Wise N:M Sparse Pattern

Compressing non-zero weights with metadata (positions of non-zero weights in the tensor) can store sparse weights more efficiently. In this subsection, we introduce various encoding schemes for sparse tensors and discuss their implications on the storage and processing requirements, i.e., on-chip decoding.

Weights with *N*:*M* sparse patterns have lower redundancy than random pruned weights. It is because the coarse-grained and fine-grained parts can be encoded individually, from which basic address and offset address are generated, respectively. The basic address indicates the order of the *M*-tile, and the offset address contains the information about the position of *N* non-zero weights inside each *M*-tile. For the randomness inside each *M*-tile, we focus on the encoding of the offset address.

Dave et al. in [19] summarized different encoding schemes. Only 1D encoding schemes will be considered for the intra-*M*-tile encoding. Coordinate (COO) stores absolute positions of non-zero weights. It does not need an extra decoding part. However, the storage for indexes may exceed that for non-zero weights when *N* is large. Run-length coding (RLC) encodes weights with the number of repetitions as ‘run’, which is the number of consecutive zeros under the sparse situation. The accumulation of ‘run’ would decode weights. Zeros will be added into the non-zero weights when the repetition exceeds the pre-defined maximum ‘max run’. Thus, the storage of RLC-encoded weights is combined with the distribution of zeros and the setting of the ‘max run’. A bitmap compresses non-zero weights with *M* one-bit flags. If the *i*-th weight in the *M*-tile is zero, the *i*-th flag is set to low. The data width of the Bitmap equals *M*. For the decoding part, an architecture proposed in [12] can decode bitmap to non-zero weight positions in the one-hot data format, which will be integrated into our decoding scheme.For the decoding, [12] provided the architecture from bitmap to non-zero weight positions in the one-hot data format.

**Figure 6:**
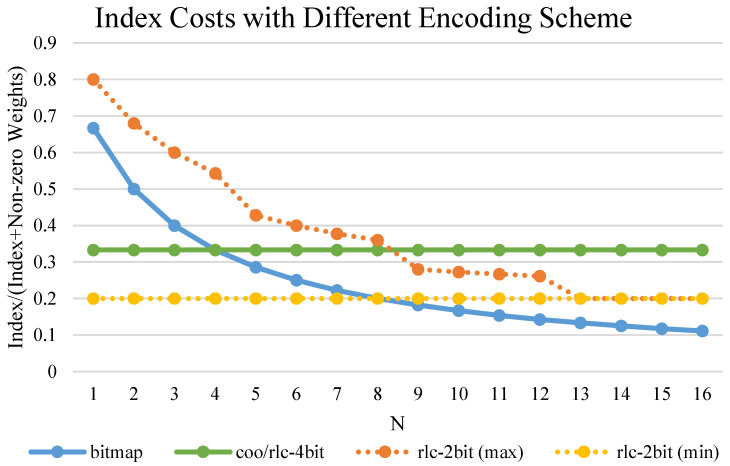
Comparison of different encoding schemes with M=16. For RLC, the ‘run length’ is 4 (2 bits), and the compact and loose distribution of zeros are shown as ‘min’ and ‘max,’ respectively.

Considering the layer-wise variable sparse pattern, we perform cost analysis for different encoding schemes. Figure 6 shows the storage costs for different coding schemes under M=16 and weight quantization bits $Q_{w}$ = 8. When N≤4, the storage of RLC with ’max run’ = 4 is minimum, while Bitmap has the minimum consumption with N≥4. Combining with the decoding costs, Table 2 represents the off-line encoding scheme utilized under different configurations of *N*. To facilitate the storage of compressed weights with different sparsity and encoding schemes, we pack 4 *M*-tile non-zero weights and indexes. In each package, indexes are packed first, followed by non-zero weights.

**Figure 7:**
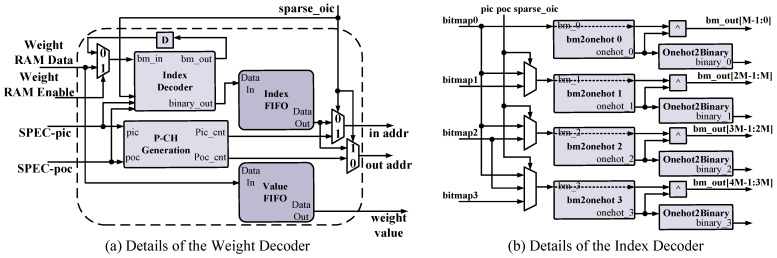
Details of the Weight Decoder. The ‘D’ in a box denotes the register used for delay.

The Weight Decoder unit in Figure 3 decodes the compressed non-zero weights. Details of the unit are shown in Figure 7a. Weight packages are fetched from global buffers. Indexes located in front of each package are decoded by the Index Decoder, which is shown as Figure 7b. The decoded binary indexes are buffered into the Index FIFO, and the bitmap after each decoding will be fed back to the Index Decoder. Non-zero weights are read after indexes in each package, which are pressed into the Value FIFO. The read control of the two FIFOs is identical. Thus, the corresponding index and non-zero weight would be fetched into Mul_Groups in Figure 2 simultaneously.

As mentioned in Section 4.1, dense dimensions are induced in SPECs for efficient processing. The sparse_oic signal indicates the sparse dimension of the SPEC. When sparse_oic is high, the output channel is *N*:16 sparse. To generate control signals for the dense dimension, the P-CH Generator unit is applied in the Weight Decoder.

The SPEC can be configured flexibly to process sparsity. Parallelisms for input-channel and output-channel inside the SPEC are denoted by pic and poc, where pic × poc = G. Examples are illustrated to explain the configuration for Weight Decoder. With sparse_oic equals to 1, addresses for PSUM and input registers are generated by Index Decoder and P-CH Generator, respectively. When poc equals 4, independent bitmaps are decoded per Figure 7b. Meanwhile, identical inputs are fetched across groups. An identical bitmap is decoded with the Index Decoder when poc equals 1. Inputs from (3,2,1,0) channels are deployed in groups. PSUMs are added across groups and accumulated with the PSUM register selected by the decoded index.

### 4.3. Overall Architecture

**Figure 8:**
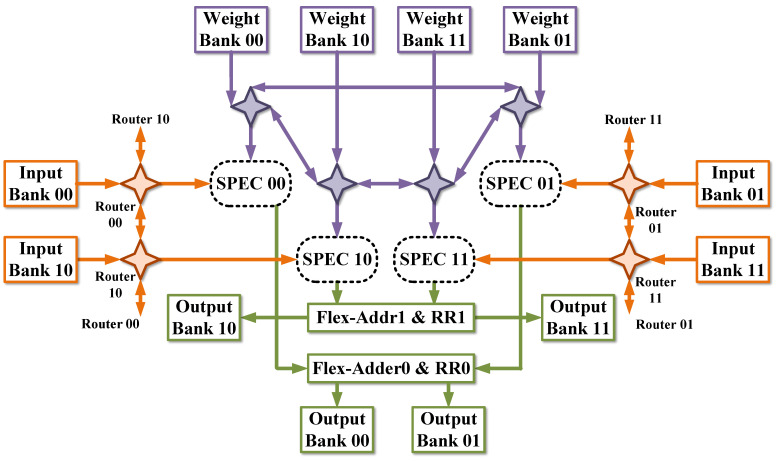
An example of an overall architecture with 2 × 2 SPECs. The RR module contains re-quantization and ReLU units. Four-pointed stars denote routers from [20].

With the proposed SPEC, the randomness of layer-wise *N*:*M* sparse pattern can be processed locally. Thus, the proposed SPEC can be integrated into any dense CNN acceleration architecture. Figure 8 shows one overall hardware architecture. It consists of data memories, computation units, and control units. Memories include input buffers, weight buffers, and output buffers. Inputs and weights are stored in the external buffer before the processing, and outputs are written back to the off-chip buffer. The external memories are connected with on-chip memories through the DMA interface. To reduce the overall processing latency, each on-chip buffer component is constructed with the ping-pong buffer structure, which can overlap the communication and calculation latency. Additionally, these buffers have multiple banks to provide sufficient bandwidth for SPECs. The connections between storage and computation units are established by routers origin from [20]. Outputs of SPECs are accumulated using ACCUs from our prior work [21]. It can process multi-level additions under configurations. In this paper, 4-in, 2-in, and 1-in additions are selected in ACCUs to accumulate PSUMs across row-wise SPECs. The mapping strategy across SPEC is determined with top controls based on the specific convolutional layer. For layers with high resolution and narrow channels, weights are broadcast across SPECs to process more data from the same feature map plane. Meanwhile, inputs are unicast across SPECs, and outputs are also unicast to output buffers, leading to the 1-in addition configuration for the ACCU module. However, for layers with wide channels and low resolution, weights are unicast across SPECs to process data across channels in parallel. Inputs and outputs are capable of getting reused in parallel. Routers and ACCUs are responsible for performing this process, which is configured by control units.Routers and ACCUs are configured with control units.

**Figure 9:**
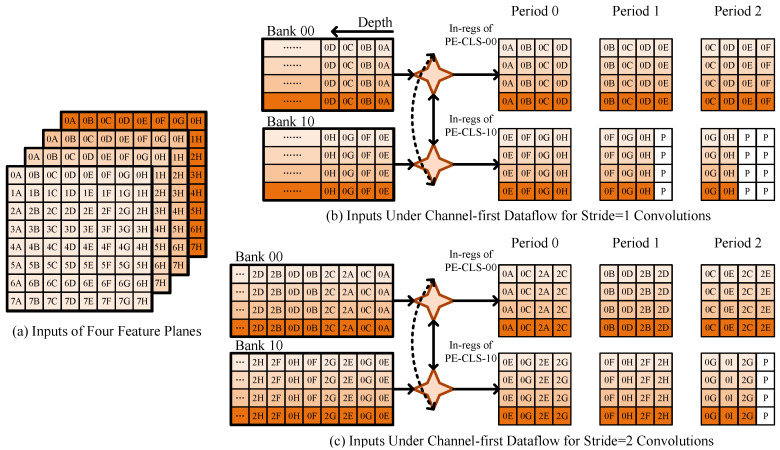
The data movement with stride = 1 and stride = 2 under the channel-first dataflow. P denotes the padding element.

Channel-first dataflow [22] was proposed to convert convolutions into GEMMs implicitly. We utilize the dataflow to facilitate the transmission between global buffers and SPEC registers. Activations from the same position across an *M* contiguous feature map plane are arranged as a word in global buffers, i.e., input banks and output banks. Pixels from the feature map plane fill in the depth of buffers. Figure 9 shows the layout of input buffers. With the channel-first dataflow, Depth-Shuffle in each SPEC obtains data to be processed regardless of convolutional stride or inter-tile overlapping.

The convolution is performed using the sliding window operation when the kernel size is not 1×1. When processing adjacent kernels, only several inputs from the same feature plane are replaced, and other data are reused in local registers. It is processed with the Depth-Shuffle unit in Figure 3. The Shuffle_mode signal is induced to indicate the renewal process, which is generated based on convolutional stride and processing parallelism. The insertion of this unit avoids repeated accesses to buffers when computing the convolution, hence reducing the overall power consumption [23].

**Figure 10:**
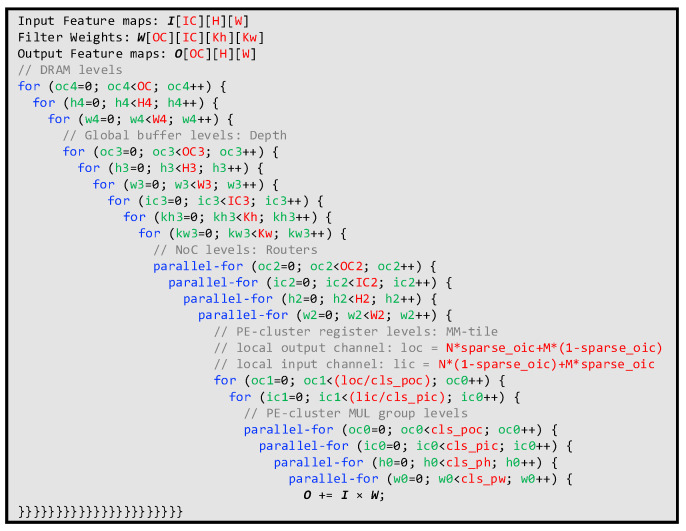
The pseudo-code for the overall dataflow.

The overall processing procedure can be summarized as follows. First, layer-wise data and configurations are transferred on-chip and stored in on-chip buffers and reconfigurable registers, respectively. Top controls of the accelerator are generated based on these reconfigurable registers. After the initial transmission, SPECs receive inputs and weights through configured routers. MM-tile PSUMs are generated in each SPEC and accumulated using ACCUs. To recover data precision, re-quantizations are performed on each PSUM. Outputs are generated after non-linear operations, such as ReLU, and fed back to output buffers. During the on-chip processing, ping-pong buffers communicate with off-chip memory simultaneously. The pseudo-code for the overall dataflow is shown in Figure 10.

## 5. Experiments and Results

### 5.1. Algorithm Evaluation

We perform *N*:*16* with 50% sparsity for Alexnet [1], Vgg-16 [2], Resnet-18, and Resnet-50 [3] on the ImageNet [14]. Comparisons of the Top-1 accuracy under different sparse pattern settings are shown in Table 3. The last layer of the classifier of all networks is dense. It can be seen that the sparsity of any dimension does not have a great impact on network accuracy. The input channel is slightly higher than the output channel in most cases due to the limited input channel of the first layer (RGB).

Thus, the pruning dimension is negligible for the deployment of algorithms. We will present accelerator performances with proposed SPECs in the next subsection.

### 5.2. Hardware

#### 5.2.1. Hardware Performances of Proposed Architectures

We evaluate our design by implementing accelerators with different SPECs. We name the accelerator processing input-channel-wise sparse, output-channel-wise sparse, and input-output-channel-wise sparse as input-channel-wise sparse architecture (ISA), output-channel-wise sparse architecture (OSA), and the input-output-channel-wise sparse architecture (IOSA), respectively. In each architecture, 16 SPECs are allocated. For ISA and OSA, only one-side channel sparse can be supported. We implement ISA on the Xilinx ZCU102 FPGA. The OSA and IOSA are evaluated on the Xilinx VCU118 FPGA. In this article, we use Verilog for RTL implementation and employ Xilinx Vivado (v2020.2) to compile the source code to the Place & Routing with the ‘Default’ strategies in both Synthesis and Implementation procedures. Further, we set the max fan-out limitation to signals manually to meet the timing constraints.

The performances of three architectures are presented as Table 4. All inputs and weights are quantized by 8-bit, and 32-bit quantization is utilized for PSUMs. To better utilize the resource on-chip, we implement our designs with different schemes. For ISA, we implement each MAC with a DSP. For OSA and IOSA, 512 out of 4096 DSPs are allocated for 1024 multiplications, where each DSP can address two 8-bit × 8-bit multiplication. Other DSPs are utilized for the accumulation in Figure 5.

The proposed I-SPEC is theoretically symmetrical to the O-SPEC. From Table 5, we can see that the resource consumption for ISA is smaller than that for OSA and IOSA, especially for logic resources. Accumulators in each SPEC for OSA and IOSA are much more complicated than in the ISA, whose origins are from three aspects. First, the compressor adders are introduced for additions across groups in each SPEC. Second, accumulations for OSA and IOSA are controlled by decoded indexes. Extra selection and enable are needed in the OSA and IOSA. Apart from the inter-group additions and weight-controlled accumulations, OSA and IOSA also have larger multiplexers. To ensure the precision accuracy, 32-bit accumulation is utilized in our accelerators for 8-bit inputs. Thus, one PSUM multiplexer is 4× more complex than one input multiplexer.

As mentioned before, different channel-wise pruned networks have similar accuracy. The ISA outperforms OSA and IOSA in hardware efficiency. Hence, the input-channel-wise sparse is more efficient for the deployment of the layer-wise *N*:*M* sparse pattern.

#### 5.2.2. Evaluation and Comparison

We compare the ISA with other accelerators, as shown in Table 5. To ensure a fair comparison, all accelerators included in our analysis are sparse and have comparable workloads, as indicated by the MAC reduction [24]. Since the fully-connected (FC) layer of ResNet-50 contributes only a small fraction to the overall computation, we compare the hardware performances of ResNet-50 with similar sparsity levels. Specifically, we compare our results for ResNet-50 with those in [21,24], both of which use 45% sparsity, by applying 50% sparsity to the network. In addition, we compare our results with those in [25] by using ResNet-50 with 25% unpruned weights.

For algorithms with similar sparsity, the accuracy after layer-wise *N*:*16* pruning is comparable to the unstructured pruned network accuracy [9]. As for hardware, the proposed ISA outperforms unstructured pruned accelerators [21,25] in processing speed and power consumption for VGG-16 and ResNet-50. When processing unstructured pruned weights, accelerators are underutilized for the load imbalance and conflict memory access due to random distributions [21]. Extra buffering and logic are implicated in alleviating the under-utilization. Besides the efficient sparse pattern, the ISA also benefits from the dedicated SPEC design and the channel-first dataflow. The SPEC eliminates the possible under-utilization by serial processing non-zero weights intra-*M*-tile with the help of the weight-broadcasting and the introduced dense dimension. Further, the channel-first dataflow and the SPEC limit sparse processing to the interior of the SPEC, eliminating the need for the overall architecture to handle sparsity.

However, for Alexnet, the first layer and FC layers account for a large percentage of overall computations, in which the ISA does not perform sparse processing. Thus, ref. [25] can process faster compared to the ISA. For output-channel-wise pruning, output channels can be partitioned into *M*-tiles and perform the layer-wise *N*:*16* pruning. After simulation, the OSA and IOSA have better speedup compared to [25] by 10% but have larger logic consumption.

For structured pruning algorithms, the layer-wise *N*:*M* pruning has a larger pruning space, which leads to better algorithm performance. The ISA can achieve much better performance compared to the structured pruned accelerator [24] for ResNet-50. It is because the 1×1 convolution performance of the architecture in [24] is bounded by the limited bandwidth. To avoid this problem, we utilize the ping-pong structure in the global buffer and the local register hierarchies. The structure is able to overlap the processing and data transmission, which shortens the overall latency. Further, the flexible network-on-chip constructed by the router and ACCUs provide sufficient design space for acceleration. Channel-first dataflow minimizes the impact of feature map size on hardware efficiency and eliminates the useless computation introduced by non-unit strides. Thus, the proposed design achieves a higher performance of power efficiency because we leverage the sparse pattern and achieve a high deployment efficiency.

## 6. Conclusions

In this article, we propose a dedicated hardware design named SPEC for layer-wise *N*:*M* sparse CNN acceleration. It can be flexibly configured to efficiently map sparse operations. Moreover, we add the sparse dimension into the pruning space. Algorithm and hardware analysis and experiments are performed regarding the enlarged pruning space. In addition, architectures with proposed SPECs are developed with flexible network-on-chip and efficient dataflow. Experiments demonstrated that our implementation could achieve up to 434-, 35-, and 150-image/s performances for AlexNet, VGG-16, and ResNet-50 on Xilinx ZCU102, respectively. Under a similar sparsity, the proposed architectures can achieve much higher power efficiency over existing sparse CNN FPGA accelerators.

## Figures and Tables

**Table 1:** The accuracy of *N*:*M* pruned networks under different configurations. Data are obtained from [8,9].

Method	Uniform	*N*:*M* Configuration	Top-1 Acc(%)	Sparsity
[8]	Dense	Dense	77.30%	0%
SR-STE [8]	Uniform	2:4	77.00%	50%
SR-STE [8]	Uniform	4:8	77.40%	50%
SR-STE [9]	Uniform	4:16	76.50%	75%
SR-STE [8]	Uniform	2:8	76.20%	75%
SR-STE [8]	Uniform	1:4	75.30%	75%
SR-STE [9]	Uniform	2:16	74.40%	87.50%
DS [9]	Layer-wise	N:16	75.70%	87.50%
SR-STE [9]	Uniform	1:16	70.70%	93.75%
SR-STE [9]	Uniform	2:32	71.50%	93.75%
DS [9]	Layer-wise	N:32	73.50%	93.75%

**Table 2:** The encoding scheme of the proposed SPEC with different values of *N* and M=16.

*N*	*N* = 1	*N* = 2	3≤N≤4	5≤N≤16
Encoding	COO	COO	COO	Bitmap

**Table 3:** Networks Performance Comparisons with *N*:16 Layer-wise Sparse Patterns.

Networks Accuracy	Alexnet	VGG-16	Resnet-18	Resnet-50
Dense	56.553%	71.082%	69.649%	76.033%
Input-Channel Sparse	55.559%	70.720%	68.935%	75.329%
Output-Channel Sparse	55.743%	70.755%	68.749%	75.327%

**Table 4:** Resource Utilization Details for Proposed Architectures.

Architectures	ISA	OSA	IOSA
FPGA Platform	Xilinx ZCU102	Xilinx VCU118	Xilinx VCU118
DSP Utilization	1024	4096	4096
	(40%)	(60%)	(60%)
Logic Utilization	500 K	600 K	645 K
	(84%)	(23%)	(25%)
BRAM Utilization ^1^	320	320	320
	(18%)	(7%)	(7%)

^1^ Number of 18 kb block ram is utilized to measure the memory consumption.

**Table 5:** Sparse Accelerator Performance Comparisons.

	[25]	Ours	[25]	Ours	[21]	[24]	Ours	[25]	Ours
CNN Type	Alexnet		VGG-16		Resnet-50				
Device	Xilinx		Xilinx		Intel	Xilinx			
	ZCU102		ZCU102		SX660	ZCU102			
Pattern ^1^	U	L	U	L	U	S	L	U	L
Sparsity (%)	10.80	37.50	11.70	37.50	45.00	45.00	50.00	23.5	25.00
MAC Reduction (%)	65.1	62.5	67.4	62.5	-	52.3	50	-	75
Frequency (MHz)	200	200	200	200	170	200	200	200	200
Precision (bits)	16	8	16	8	8	16	8	16	8
DSP Utilization	1144	1024	1144	1024	512	1344	1024	1144	1024
LUT Utilization	552 K (92%)	500 K (84%)	552 K (92%)	500 K (84%)	102.6 K (41%)	390 K (65%)	500 K (84%)	552 K (92%)	500 K (84%)
BRAM Utilization ^2^	912 (48%)	320 (18%)	912 (48%)	320 (18%)	465 (22%)	1460 (80%)	320 (18%)	912 (48%)	320 (18%)
Performance (image/s)	446	434	31	35	23	57	**83**	149	**150**
Power (W)	23.5	15.0	23.5	15.0	4.6	15.4	15.00	23.5	15.00
Power Efficiency	18.98	**28.93**	1.32	**2.33**	5.00	3.70	**5.53**	6.34	**10.00**

^1^ U denotes unstructured pruning. S denotes structured pruning. L denotes layer-wise *N*:16 pruning. ^2^ Numbers of 18 kb block ram and 20 kb block ram are utilized to measure the memory consumption for Xilinx FPGA and Intel FPGA, respectively.

## Data Availability

Not applicable.
